# Mammalian Glutaminase *Gls2* Gene Encodes Two Functional Alternative Transcripts by a Surrogate Promoter Usage Mechanism

**DOI:** 10.1371/journal.pone.0038380

**Published:** 2012-06-05

**Authors:** Mercedes Martín-Rufián, Marta Tosina, José A. Campos-Sandoval, Elisa Manzanares, Carolina Lobo, J. A. Segura, Francisco J. Alonso, José M. Matés, Javier Márquez

**Affiliations:** Laboratorio de Química de Proteínas, Departamento de Biología Molecular y Bioquímica, Facultad de Ciencias, Universidad de Málaga, Málaga, Spain; Consejo Superior de Investigaciones Cientificas, Spain

## Abstract

**Background:**

Glutaminase is expressed in most mammalian tissues and cancer cells, but the regulation of its expression is poorly understood. An essential step to accomplish this goal is the characterization of its species- and cell-specific isoenzyme pattern of expression. Our aim was to identify and characterize transcript variants of the mammalian glutaminase *Gls2* gene.

**Methodology/Principal Findings:**

We demonstrate for the first time simultaneous expression of two transcript variants from the *Gls2* gene in human, rat and mouse. A combination of RT-PCR, primer-extension analysis, bioinformatics, real-time PCR, *in vitro* transcription and translation and immunoblot analysis was applied to investigate GLS2 transcripts in mammalian tissues. Short (LGA) and long (GAB) transcript forms were isolated in brain and liver tissue of human, rat and mouse. The short LGA transcript arises by a combination of two mechanisms of transcriptional modulation: alternative transcription initiation and alternative promoter. The LGA variant contains both the transcription start site (TSS) and the alternative promoter in the first intron of the *Gls2* gene. The full human LGA transcript has two in-frame ATGs in the first exon, which are missing in orthologous rat and mouse transcripts. *In vitro* transcription and translation of human LGA yielded two polypeptides of the predicted size, but only the canonical full-length protein displayed catalytic activity. Relative abundance of GAB and LGA transcripts showed marked variations depending on species and tissues analyzed.

**Conclusions/Significance:**

This is the first report demonstrating expression of alternative transcripts of the mammalian *Gls2* gene. Transcriptional mechanisms giving rise to GLS2 variants and isolation of novel GLS2 transcripts in human, rat and mouse are presented. Results were also confirmed at the protein level, where catalytic activity was demonstrated for the human LGA protein. Relative abundance of GAB and LGA transcripts was species- and tissue-specific providing evidence of a differential regulation of GLS2 transcripts in mammals.

## Introduction

The biological diversity of proteins in higher eukaryotes can be increased by different processes operating at the transcriptional level, where alternative splicing, alternative transcriptional initiation, alternative polyadenylation, alternative promoters and RNA editing represent the major mechanisms allowing the generation of surprisingly great proteome diversity in higher eukaryotes despite the relatively small gene number. Alternative transcription initiation sites and alternative splicing are two well characterized mechanisms providing transcript diversity [1], [2]. Increasing evidences support that most human genes present transcript variants arising by one or combination of several transcriptional regulation mechanisms. Thus, large-scale characterization of human transcriptome indicates that between 69–75% of genes undergo alternative splicing [3], [4], 81% are subject to alternative transcription initiation [5], 60% to alternative polyadenylation [6], and 52% were subject to regulation by putative alternative promoters [7]. Furthermore, investigations in recent years have demonstrated the importance of transcript variant expression to explain developmental changes, cell-specific differentiation processes and metabolic deregulations conducive to pathological states [8], [9].

Glutaminase (GA^1^; E.C. 3.5.1.2) plays essential roles in cell bioenergetics and nitrogen metabolism by converting glutamine into glutamate plus ammonium ions. Two different genes in distinct chromosomes code for mammalian GA enzymes: the *Gls* gene codes for kidney-type (K-type) isozymes, whereas the *Gls2* gene encodes liver-type (L-type) isozymes [10]. Two K-type transcripts have been described: the canonical KGA transcript originally found in kidney and composed by 18 exons [11], and the alternative spliced form named GAC, first described in human kidney and colon cancer cells, which contains only 15 exons [11], [12]. On the other hand, nothing is known about the expression and processing of L-type transcripts arising from the *Gls2* mammalian gene. The first L-type GA transcript, named LGA, was cloned from rat liver and originally thought to be present only in adult liver tissue [13], [14]. Nevertheless, a second L-type GA mRNA cloned from ZR-75 human breast cancer cells [15], and later termed GAB [16], was demonstrated to be also expressed in extrahepatic tissues, like brain, pancreas, human cancer cells and cells of the immune system [15], [17], [18], [19].

Glutaminases have attracted considerable attention in last years because of the discovery of novel interacting partners and subcellular locations which strongly suggest they behave as multifunctional proteins, besides their roles as classical metabolic enzymes. In tumor biology, a correlation between GA expression and cancer growth and proliferation was early established, supported by an enhanced mitochondrial glutamine catabolism frequently exhibited by many types of tumor cells [20], [21], [22]. Recently, GA have fuelled attention in cancer research because of key discoveries revealing their involvement as targets of oncogenes and tumor suppressor genes. Thus, expression of human *GLS* gene has been associated to oncogene c-Myc through a miRNA mechanism allowing overexpression of K-type GA proteins in parallel with cancer cell proliferation [23]. On the other hand, *GLS2* was demonstrated to be target of the p53 tumor suppressor gene as mediator of p53′s role in energy metabolism and antioxidant defense [24], [25]. A differential function of GA proteins was first suggested by our group after examination of their pattern of expression in several cancer cell lines and human tumors: GLS isoforms were associated to tumorigenesis while GLS2 were ascribed to cell differentiation [18]. It is presently unknown how GA isoenzymes may undergo such different roles in tumor biology [26].

Furthermore, some mammalian tissues and tumor cells show co-expression of GLS and GLS2 isoforms [10], [18], although the physiological meaning of the existence of different GA transcripts is not understood [17]. For example, we described a simultaneous and differential expression of GLS and GLS2 proteins in mammalian brain [27], in sharp contrast to the classical view which postulated that KGA was the unique isoform expressed in brain. The discovery of new tissues and cells expressing GLS2 proteins, along with novel characteristics and functions recently suggested for GLS2 isoforms [28], [29], call for a characterization of different transcripts encoded by the mammalian *Gls2* gene, as a prerequisite to understand tissue-specific regulation of GA and glutamine metabolism.

In this study, we use a combination of biochemical, molecular biological, and bioinformatics approaches to show for the first time direct evidence of the existence of alternative mRNA forms of the orthologous *Gls2* gene in mouse, rat and human. We conclude that there are, at least, two sense transcripts encoded by the *Gls2* gene: a long canonical transcript containing all 18 exons of the CDS, termed GAB, and a short transcript variant, LGA, which appears by alternative transcription initiation. LGA variant uses an alternative promoter located more than 7 kb apart from the canonical GAB promoter, and has a distinct alternative first exon. Interestingly, GAB and LGA are differentially expressed in mammalian tissues and human cancer cells.

## Materials and Methods

### Ethics Statement

Rats were maintained in the Central Animal Housing Facility of the University of Malaga at the Faculty of Medicine. The maintenance of the animals as well as the experimental procedures were in accordance with the European animal research laws (European Communities Council Directives 86/609/EU, 98/81/CEE, 2003/65/EC and Commission Recommendation 2007/526/EC). Furthermore, all experimental protocols dealing with animals here reported were previously approved by the Animal Care Panel of the Center for Animal Experimentation of the University of Málaga.

### Materials

Brain and liver poly(A)^+^ mRNA from human and mouse were purchased from Clontech. Male Wistar rats (9–10 weeks old) were killed by decapitation and their tissues quickly removed and frozen in liquid nitrogen. Rat liver and brain total RNA were isolated using the RNeasy Mini Kit® (Qiagen); DNase digestion was completed during RNA purification using the RNase Free Dnase Set (Qiagen), according to the manufacturer’s instructions. Total RNA integrity was checked by agarose gel electrophoresis and quantified using the 260 nm absorbance reading. RNA purity was established by calculating the ratio of the absorbance readings at 260 and 280 nm.

### Reverse Transcription (RT)-PCR Analysis

A universal RT primer previously used for cloning human GAB, 5′-CCGTGGGTCTAACTTCCGAGCAC-3′, was employed for reverse-transcription of RNA isolated from the three species tested (mouse, rat and human). Reverse transcriptase-polymerase chain reaction (RT-PCR) was carried out using 1 µg of total RNA or 0.5 µg of poly(A)^+^ mRNA. RNA was converted to first-strand cDNA using primer RT (2 µM) with SuperScript II reverse transcriptase (GIBCO/BRL) and dNTP’s (10 mM each) following standard procedures. For isolation of the full LGA transcript from human poly(A^+^)mRNA, retrotranscription was done using a oligo(dT) primer at 10 µM concentration. The cDNA was then treated with 2 units of RNaseH for 20 min at 37°C. For PCR amplification, 2 µl of cDNA reaction mixture were combined with Advantage cDNA polymerase (Clontech) and the respective primers at 500 nM. The sense (forward) primers employed were: Intron1human, 5′-ATCAGATGGCTTAAGGAGGAGGC-3′, for partial amplification of the short L-type (LGA) transcript from human liver and brain; Exon1rat, 5′-ATGCGCTCCATGAAGGCTCTG-3′, for amplification of the long L-type (GAB) transcript from human liver and brain, rat liver and mouse liver and; Intron1mouse, 5′-GCCAGATGGTTCAAAGAGGAGG-3′, for amplification of the short L-type (LGA) transcript from rat brain and mouse liver and brain. In all cases, the antisense (reverse) primer was GSP-6, a universal oligonucleotide deduced from the human GAB isoform: 5′-GCAGTGGTGAACTTGTGGATAGGG-3′. Reactions were carried out in a DNA Thermocycler iCycler (Bio-Rad) with the following parameters: 1 cycle at 94°C for 5 min; 30 cycles at 94°C for 1 min, 57°C–61,5°C (depending on the forward primer used) for 45 s, 72°C for 2 min, and a final elongation step at 72°C for 7 min. For isolation of the full LGA transcript from human brain, the primers employed were: hLGAfw: 5′-GAGGACACTCACCTACTTATAAGCCC-3′ and hLGArev: 5′-CTTCTCTGTACTCTGTCTGCTGAGG-3′, and the PCR programme was as follows: 1 cycle at 94°C for 1 min; 35 cycles at 94°C for 20 s, 55°C for 30 s, 68°C for 2 min, and a final extension at 68°C for 3 min.

### Primer Extension Analysis

The hLGA cDNA clone obtained by RT-PCR was exploited to design primers to determine the transcription start site. Three primers were deduced from the first 400 nt of the hLGA cDNA: anti-sense primers GSP-6, PEXT1 (5′-CAGAGAATGGGGAGGAAAGTG-3′) and PEXT2 (5′-CATGGGCTTATAAGTAGGTGAGTG-3′). The oligonucleotides were ^32^P end-labelled with [γ-^32^P]ATP and T4 polynucleotide kinase. Poly(A)^+^ mRNA of human brain (approx. 0.5–1 µg) was mixed with the radioactive oligonucleotides and reverse transcribed with avian myeloblastosis virus reverse transcriptase, with the use of the protocol provided by the manufacturer (Primer Extension Analysis kit; Promega). A kanamycin-positive control RNA was run in parallel and the primer extension products were analyzed on denaturing polyacrylamide analytical gels containing 8% (w/v) polyacrylamide, 7M urea and 1xTBE (Tris-borate-EDTA) buffer. Negative controls with mRNA omitted were always run in parallel. Gels were dried in a gel dryer at vacuum and revealed by autoradiography. The size of the single-stranded cDNA products (in bases) was estimated approximately by comparison with ФX174 HinfI DNA markers, using Image Lab software and a Pharos FX Molecular Imager (Bio-Rad).

### Western Assays and Analytical Determinations

Mitochondria from mouse liver and brain were isolated as previously described [30]. Mitochondrial fractions were analyzed by SDS-PAGE and Western blotting essentially as described [29]. Isozyme-specific antibodies against human L-type GA (GAB+LGA) and KGA were used as described before [27], [30]. A new polyclonal monospecific antibody was generated against the human GAB isoform. This GAB-specific antibody was raised against the synthetic peptide: RETPHSHQPQHQDH belonging to exon 1 of human GAB and, therefore, not contained in the LGA amino acid sequence. Antibodies were commercially generated in rabbit after being crosslinked to keyhole limpet hemocyanin (Genosphere Biotechnologies, France). The blots were developed with the enhanced chemiluminiscence technique as recommended by the supplier (Amersham Biosciences). Protein content was determined by the Bradford method [31]. GA activity was measured as described elsewhere [29] with minor modifications, by measuring the ammonium released in the GA reaction with the OPA reagent. After *in vitro* transcription and translation, the reticulocyte lysate was diluted into a small volume of GA activity buffer (150 mM potassium phosphate, 1mM ammonim chloride, pH 8.0). Then, the soluble extract was concentrated to 25 µl by ultrafiltration at 11,000× g using Centricon YM-30 tubes (Millipore), and submitted to GA activity assay. The pGEM-T[hLGA] construct cloned in the reverse (antisense) orientation was always included as a control for zero background activity. After GA reaction and before spectrophotometric determinations, extracts were treated with 20% TCA to reduce background absorbance due to hemoglobin and some other components of the reticulocyte lysates.

### Expression Analyses by Real-time RT-PCR

Total RNA (1 µg) or poly(A)^+^ mRNA (500 ng) were reverse transcribed using the Quantitec Reverse Transcription kit (Qiagen) in a 20 µl reaction, according to the manufacturer’s instructions. RNA samples were tested for genomic DNA contamination by including no enzyme reverse transcription (RT) controls, and for reagent and aerosol contamination by including two no template controls. Real time PCR was carried out with the CFX thermocycler (Bio-Rad). The rat 60 s ribosomal protein L19 (Rpl19) and the mouse β-actin were used to normalize experimental variability. The partial Rpl19, β-actin, KGA, GAB and LGA transcript sequences were amplified using isoform-specific primers (Table S1). Reaction efficiency was established for each set of primers after quantification of eight different dilutions of the DNA pool. Reactions were set up in a total volume of 25 µl using 25 or 50 ng of cDNA, 2x Perfecta SYBR-Green Supermix (Quanta Biosciences) and 200 nM of each transcript-specific primer. The cycling conditions were: 95°C for 4 min, 40 cycles of 95°C for 15 s, 61°C (mouse) or 63°C (rat) for 45 s and 72°C for 30 s. Fluorescence acquisition was carried out at 72°C. Specificity of the PCR products was confirmed by analysis of the dissociation curve. Each run included the RT-negative and non-template control. Results were evaluated using CFX Bio-Rad Analysis Software. Amplicon size and the absence of non-specific products were confirmed by agarose gel electrophoresis. For absolute quantification of both constitutive and GA transcripts, PCR transcript-specific amplicons were prepared as a standard. PCR products were analysed by 2% agarose gel electrophoresis and purified (Illustra™ GFX™PCR DNA and Gel Band Purification Kit, GE Healthcare). Concentrations of purified nucleic acids were determined by UV absorbance spectrophotometry. Tenfold serial dilutions of the quantified amplicon solutions were kept in aliquots at −20°C and used throughout the study as external standards of known concentration for the real time PCR reactions (range of standards: 10^2^–10^−5^ pg).

The standard curve was created by plotting the threshold cycle (Ct) corresponding to each standard, *vs* the value of their corresponding log of starting quantity (pg). Ct is the number of required cycles to reach the defined threshold level. Results in liver and brain of the different GA transcripts were expressed as numbers of molecules (±SEM) of mRNA per ng of total RNA or mRNA, corrected for Rpl19 or β-actin constitutive values. Absolute transcript copy number was calculated by using the Avogadro constant (6.023×10^23^) and its molecular mass [32].

### In vitro Transcription and Translation

The RT-PCR product of 1900-bp of human LGA cDNA was cloned into the pGEM-T vector. This insert encompasses the complete coding sequence, beginning 3 bp upstream of the start codon and including 26 bp of 3′-untranslated region. Human LGA transcript variants lacking exon 4 and exon 10 were cloned into pGEM-T and included as controls, as well as hLGA cloned in the antisense orientation. We also prepared a deletion mutant construct of human LGA starting at the second ATG codon of the full transcript. Purified plasmids were transcribed and translated using the TNT™-coupled reticulocyte–lysate system according to the manufacturer’s instructions (Promega), using 1 µg of recombinant DNA and 20 µCi of [^35^S]-Met in a total volume of 50 µl. After 60-min incubation at 30 °C, aliquots were removed for further experiments. Unlabeled methionine was added to the extract used for GA activity assay. Expression reactions carried out in parallel with 1 µg of the pGEM-T empty vector were used as blanks. *De novo* synthesis of polypeptides was monitored by SDS/PAGE and autoradiography. The films were analyzed with a GS800 scanner employing Quantity One software (Bio-Rad).

## Results

### Isolation of Alternative Transcripts of *Gls2* Gene

**Figure 1:**
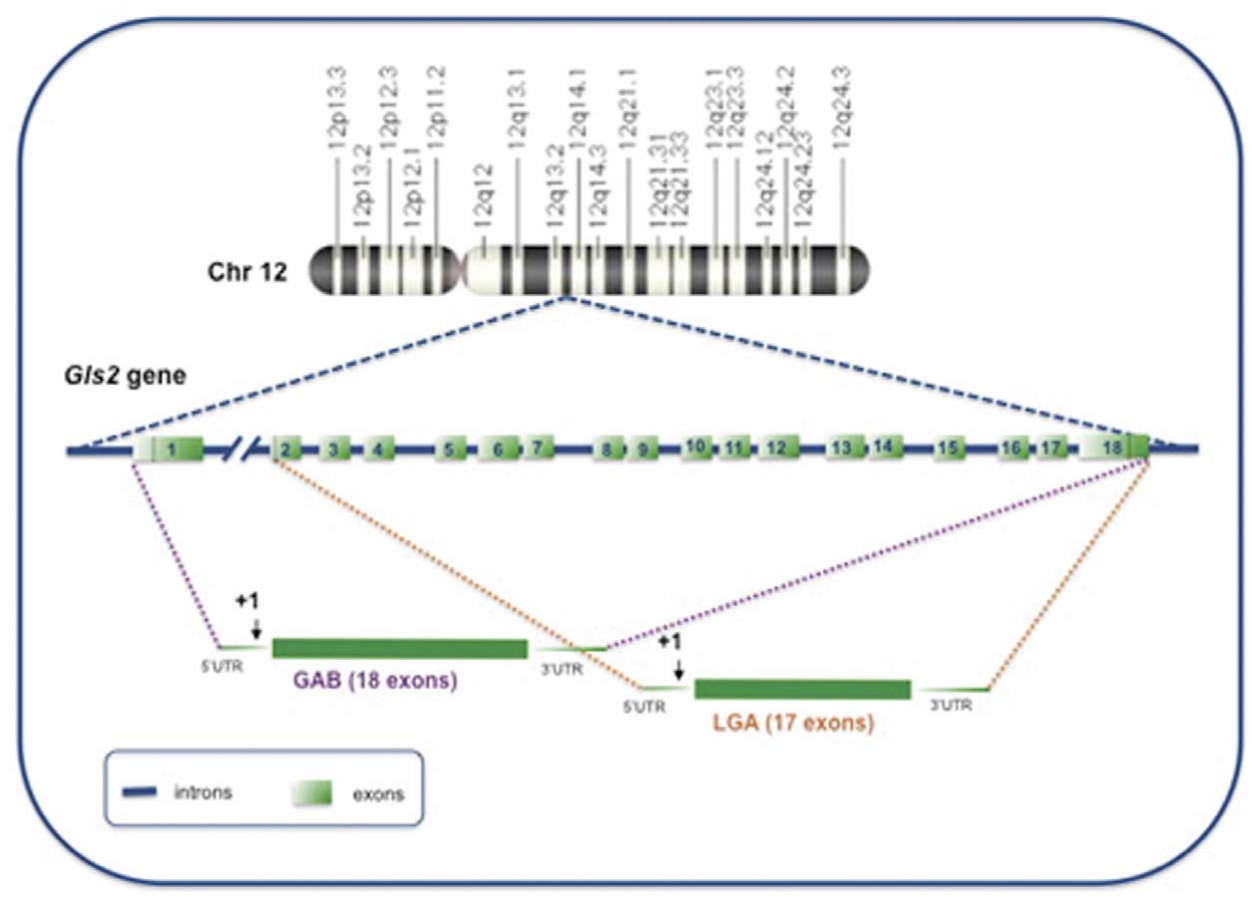
Structure of the human *GLS2* gene and predicted GA transcripts. The GLS2 gene is located in the 12q13 region of human chromosome 12, as shown in the upper part of this figure. The gene has a length of approximately 18 kb and split into 18 exons. Exon sequences are indicated as numbered light green boxes; intron sequences are shown as solid blue lines. Two GA transcripts are encoded by GLS2: the canonical GAB mRNA formed by joining the full 18 exons of the gene and the short LGA transcript that lacks exon 1. Dotted lines comprise exons involved in the generation of both transcript variants. The transcription start site is marked by an arrow and numbered as +1.

The first indication of the existence of transcript variants for the mammalian *Gls2* gene (Fig. 1) came about from sequence analysis of early genome draft sequences deposited in Rat Genome Sequencing Consortium, GenBank and Ensembl databases of rat and mouse. We notice that nucleotides at the 5′-UTR and initial coding sequence of rat liver LGA [33] matched with sequences located at the 3′-end of intron 1 of *Gls2* in both human and mouse genomic DNA, suggesting that a short LGA transcript might also be expressed in these species. Therefore, RT-PCR experiments were planned in human and mouse tissues to isolate short LGA transcripts orthologous of the rat liver LGA [13].

**Figure 2:**
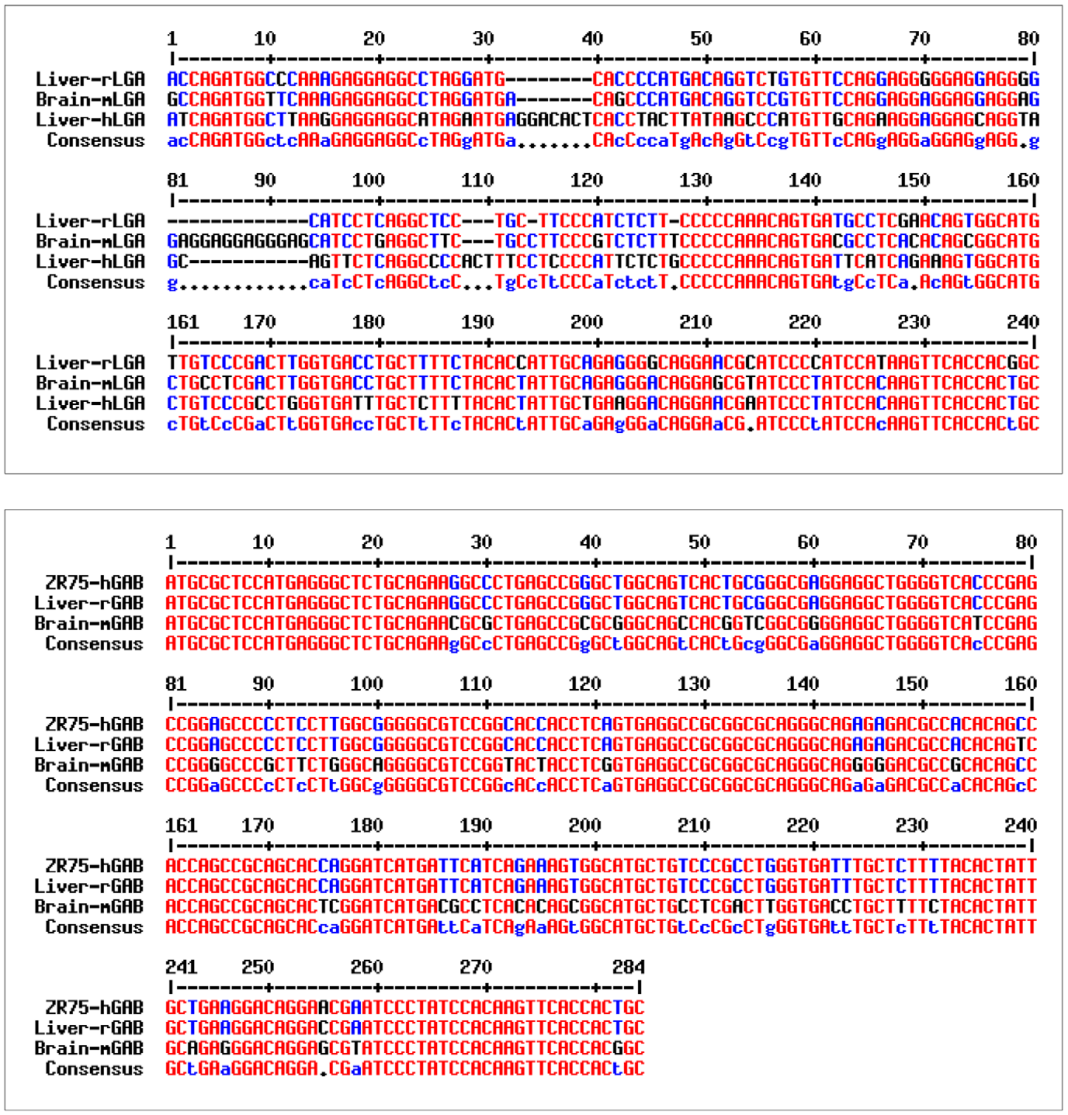
Comparison of the sequences of RT-PCR products demonstrating co-expression of LGA and GAB transcripts in mouse, rat and human tissues. The identity of each amplified fragment was assessed by sequence alignment with known sequences for rat liver LGA (GenBank# J05499) and human GAB (GenBank #AF348119) using Blast program. Top panel: the 5′-sequences of mouse brain and human liver LGA cDNA fragments obtained by RT-PCR were aligned with the sequence of rat liver LGA cDNA; bottom panel: the 5′-sequences of rat liver and mouse brain GAB cDNA fragments obtained by RT-PCR were aligned with the human GAB sequence from ZR-75 breast cancer cells. Identical nucleotides are indicated in red, different nucleotides are labeled in blue. Sequence alignment was done using Multalin program (http://multalin.toulouse.inra.fr/multalin/).

For isolation of LGA transcripts by RT-PCR, sense oligonucleotides were designed with sequences corresponding to the 3′-end of intron 1 of human [34] and mouse (NCBI *Mus musculus* Genome Build 37.2) *Gls2* genes. The human intron 1 primer was used for amplification of the human LGA transcript in brain and liver, whereas the mouse intron 1 primer was used indistinctly for mouse and rat LGA amplifications. In all cases, the antisense primer GSP-6, which anneals to exon 2 of human *GLS2* gene and to exon 1 of mouse and rat genes, was employed. PCR products of the expected lengths and with highly similar sequences to that of rat liver LGA (Fig. 2, top panel) were obtained, thus getting proof of the expression of LGA transcript forms in tissues and species distinct from rat liver. Furthermore, BLAST analysis of exon 1 of human GAB mRNA [10], [34] against rat (Rnor 3.3, Rat Genome Sequencing Consortium) and mouse (Ensembl v35) genomic sequences identified a homologous region (>95% identity) in the 5′-flanking region of both *Gls2* genes, about 5.2 kb and 4 kb upstream of their respective transcription start sites (TSS) in rat chromosome 7 and mouse chromosome 10. Therefore, a RT-PCR strategy was designed to proof the existence of orthologous GAB transcripts in liver and brain from rat and mouse. The isolated RT-PCR products (Fig. 2, bottom panel) display great homology with the human GAB cDNA cloned from ZR-75 breast cancer cells [15], proving that GAB transcripts are indeed expressed by rat and mouse.

**Figure 3:**
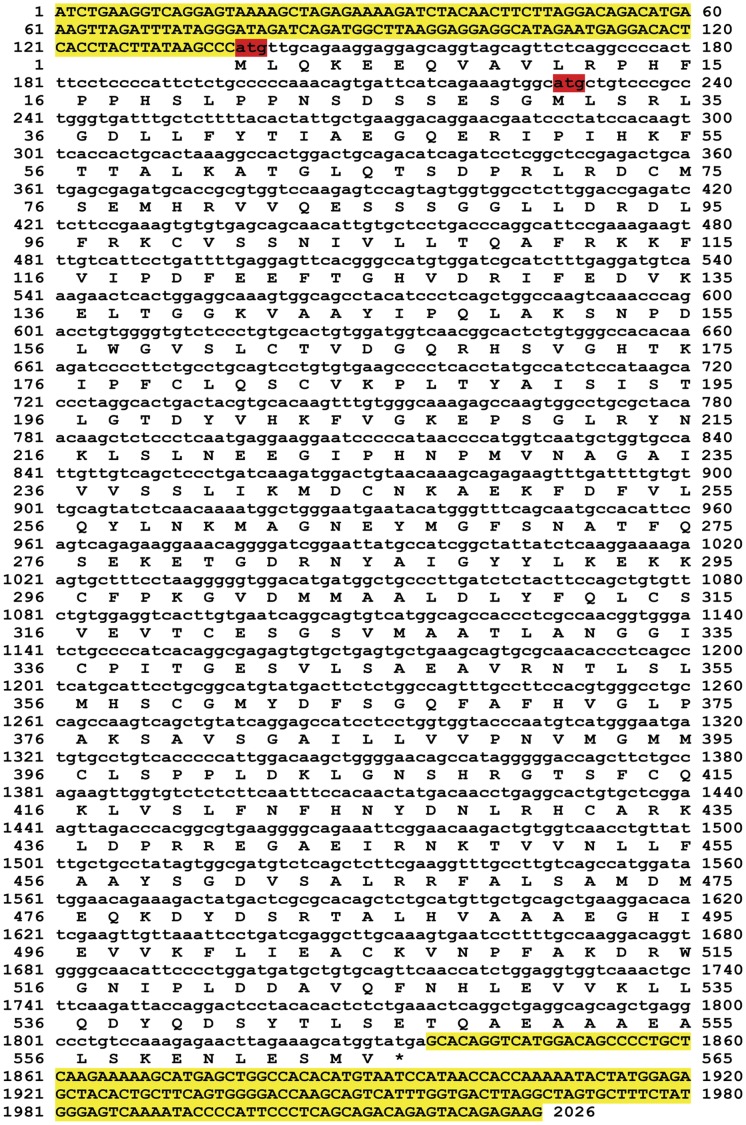
Nucleotide and deduced amino acid sequences of human LGA transcript. The short LGA transcript variant was cloned by RT-PCR from human brain mRNA as described in the Materials and Methods section. The cloned cDNA was fully sequenced: nucleotide and deduced amino acid sequences are shown. The two initials ATG start codons are labeled in red; 5′-UTR and 3′-UTR regions are marked in yellow. Amino acid 1 is the initiation methionine and the stop codon is indicated by the asterisk. Sequences were arranged using Prettyseq program (http://emboss.sourceforge.net/apps/release/6.4/emboss/apps/prettyseq.html).

Once we showed co-expression of GLS2 transcripts in mammalian tissues, thereby demonstrating that human GAB is not the orthologous transcript of rat liver LGA, we cloned the full length LGA transcript from human brain for further characterization and functional studies. The transcript was fully sequenced (GenBank# 775422, Fig. 3) and its sequence compared with rat liver LGA cDNA. Overall, human LGA nucleotide sequence shows an 89% identity with rat liver LGA (BLAST analysis); it has a 1698 nt ORF encoding a putative protein of 565 amino acids (Fig. 3). The deduced amino acid sequence was also highly similar, with a 91% of identity with regard to both rat LGA and human GAB proteins (Clustalw analysis) (Figure S1).

Three further non-coding GLS2 transcripts, containing premature stop codons, were isolated during cloning studies of LGA (results not shown). Two of them were non-sense human brain transcripts lacking exons 4 and 10, respectively (GenBank# HM775423 and HM775424). The missing exons altered the reading frame giving rise to premature stop codons. The third non-sense transcript was cloned from mouse brain by RT-PCR; in this case, a partially-retained intron 2 was present in the sequence giving rise to a non-sense transcript (GenBank# EU770627). Finally, we also identified expressed-sequence tags (EST’s) supporting *Gls2* GAB transcripts. The human GAB genomic sequence [34] was screened against the human EST database. The same analysis was made with the rat and mouse *Gls2* gene sequences deposited in the current version of the NCBI rat genome project (v37) and Ensembl databases, respectively. Several EST supported the existence of GAB long transcripts in each species: GenBank #BC104712 and #BC089776 in rat; GenBank #AI195692.1, #BB633707.1 and #BY243446.1 in mouse. In contrast, we did not find any EST for LGA transcripts in human, mouse and rat EST databases. The absence of EST LGA-specific may be due to the fact they must reach the distinct 5′-UTR sequence of LGA, a GC-rich region hard to transcript.

### Transcript Variant LGA Appears by Alternative Transcription Initiation and has Alternative Promoter

**Figure 4:**
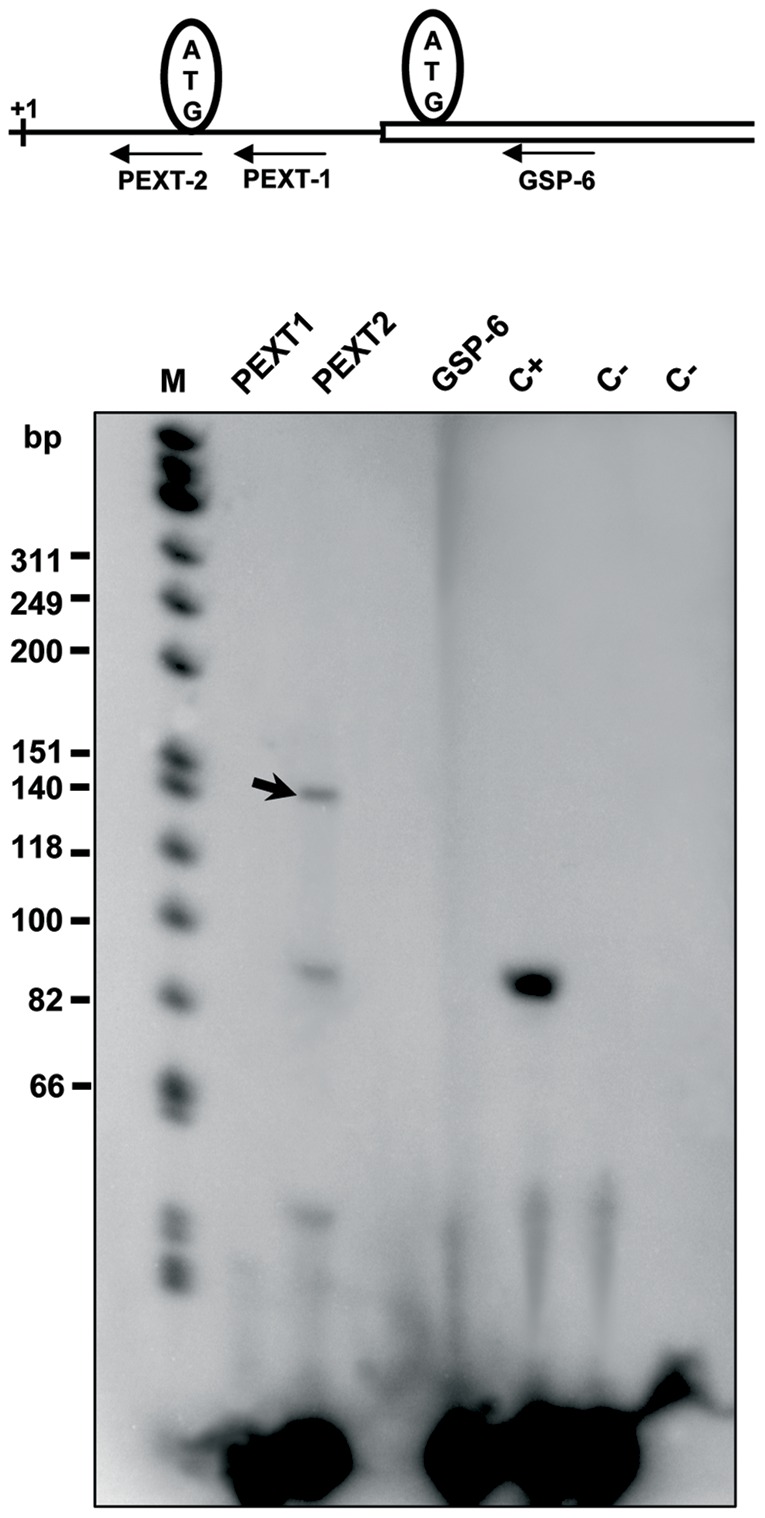
Primer extension analysis of human brain RNA. Oligonucleotides GSP-6, PEXT1 and PEXT2 flanking the 5′ termini of the human LGA cDNA clone were ^32^P end-labeled with [γ-^32^P]ATP and T4 polynucleotide kinase. Poly(A)^+^ mRNA of human brain (approx. 0.5–1 µg) was mixed with the radioactive oligonucleotides and reverse-transcribed with avian myeloblastosis virus reverse transcriptase; the products from primer extension reactions were separated by electrophoresis on an 8% (w/v) denaturing polyacrylamide gel. Lanes are labeled with the name of the specific primer used in each case. The size of the single-stranded cDNA products (in bases) was estimated approximately by comparison with ФX174 HinfI DNA markers shown at the left in lane M. C+ lane: product amplified with a positive control of kanamycin. C- lanes: negative controls using PEXT2 with mRNA omitted. The black arrow indicates the specific GA products of approx. 140 bases obtained by reverse transcription with primer PEXT2. Top panel: scheme showing the approximate position of the three primers with regard to the TSS (+1) and the two first ATG codons of human LGA transcript.

Sequence analysis of GAB and LGA variants clearly supports that LGA short transcript appears by alternative transcription initiation, because its TSS was located at the 3′-end of the first intron of the *Gls2* gene. Taking into account the length of this intron (more than 7 kb in human, >4 kb in rat and mouse), an alternative promoter, different from the one used by GAB, must necessarily exist in this 5′-flanking region. To map this regulatory region, we performed primer extension analysis to exactly localize the TSS of human LGA (Fig. 4). Although three different primers were employed, only one of them was able to prime consistently the reverse transcriptase enzyme, giving rise to two bands in the autoradiography (Fig. 4). The upper band has an apparent molecular mass of 140 nts by comparison with standard molecular markers ran parallel in the same gel. This means that human LGA transcription starts at the adenine nucleotide located 140 nts upstream of the translation start codon (Fig. 3). The sequence of the TSS conforms to consensus sequences for eukaryotic transcription starts and is almost identical to the transcription initiation site of the orthologous rat liver LGA mRNA [33].

**Figure 5:**
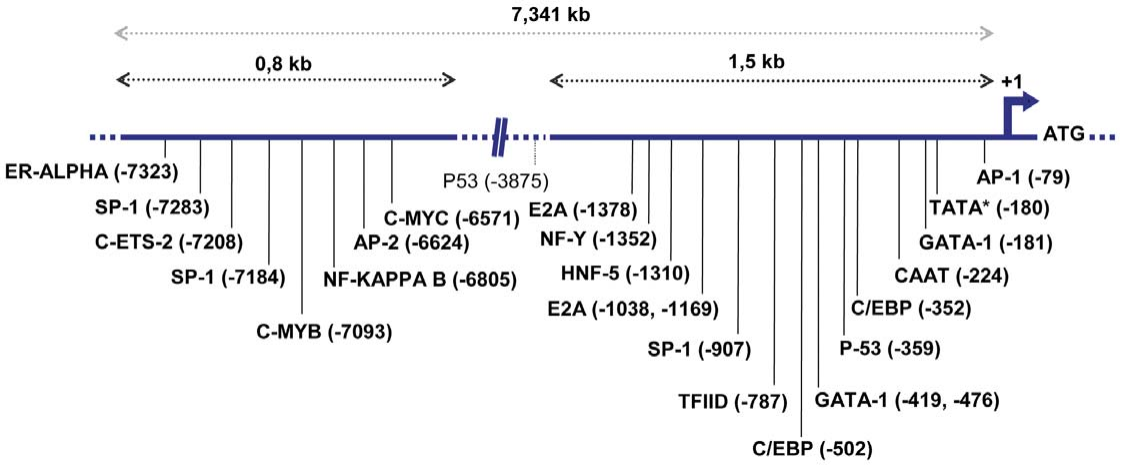
Main transcription factor recognition sites presented in the alternative promoter of the human LGA transcript. Schematic diagram showing the 7,341 kb of intron 1 of the human *GLS2* gene. The transcription start site is indicated by +1. The sequences from nt -7340 to nt -6540 (enhancer distal region) and from nt -1500 to nt +1 (proximal promoter) are represented. Main putative protein binding sites, determined by computer analysis, are identified by vertical bars and are named in bold letters below the sequence. The program TESS (http://www.cbil.upenn.edu/tess) was employed for searching consensus transcription factor motifs. A second p53 motif, found in between distal and proximal promoters, is shown in normal type characters.

Obviously, the most plausible candidate for alternative promoter of the human LGA transcript is the sequence upstream of its TSS at the 3′-end of intron 1. Bioinformatics analysis of the approximately 1,5-kb sequence 5′ distal to the transcription initiation supported this functional role: many transcription factor recognition sites are present in this region (Fig. 5), strongly suggesting that this is a functional promoter for LGA. Main transcription boxes found were AP-1, TATA-like, GATA-1, CAAT, SP1 and p53. Interestingly, a distal promoter region was also predicted for the first kb of intron 1, almost 7 kb away from the TSS, which contains c-Myb, c-Myc, AP-2, NF-kappa B and some other consensus boxes (Fig. 5).

**Figure 6:**
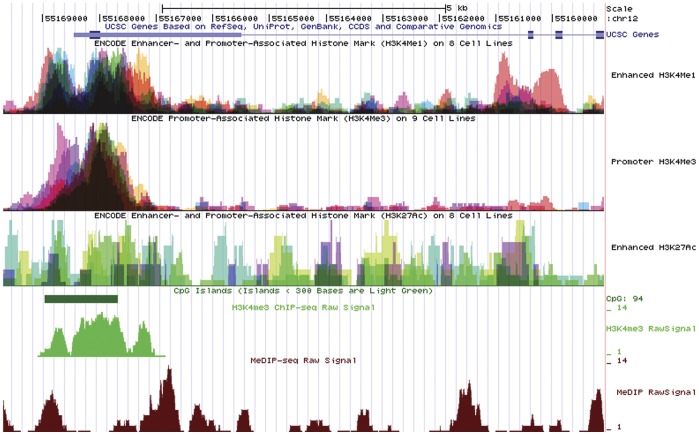
ENCODE Enhancer- and Promoter-Associated Histone Marks, CpG islands and methylation status of the *GLS2* gene locus on human chromosome 12. For simplicity, only the four first exons of human *GLS2* gene and the 5′-flanking genomic region are shown on top of the Figure. Below the *GLS2* gene, six plots are represented, as follows: first panel: Enhancer- and Promoter-Associated Histone mark (H3K4Me1) from 9 human cell lines; second panel: Promoter-Associated Histone mark (H3K4Me3) from 8 human cell lines; third panel: Enhancer- and Promoter-Associated Histone mark (H3K27Ac) from 8 human cell lines; fourth panel: CpG island shown as a solid green bar; fifth panel: Promoter-Associated Histone mark (H3K4Me3) from chromatin immunoprecipitation of DNA and sequencing data (ChIP-seq) and; sixth panel: Methylation-dependent immunoprecipitation of DNA and sequencing data from human brain (MeDIP-seq).

Converging evidence supporting the 3′-end of intron 1 as an alternate promoter which regulates LGA transcription was also obtained from the Encyclopedia of DNA elements (ENCODE) produced by the ENCODE Consortium (The ENCODE Project Consortium 2007). Analysis of the human *GLS2* genomic locus for the presence of enhancer- and promoted-associated histone marks (H3K4Me1 and H3K27Ac) in several human cell lines detected three main regions: the GAB canonical promoter, the alternative LGA promoter and a distal region located at the 5′-end of intron 1 (Fig. 6). However, the H3K4Me3 track, a histone mark associated with promoters, only detected the GAB promoter and the enhancer region at the beginning of intron 1, both in human cell lines and frontal cortex (Fig. 6). The presence of CpG islands differs notably in both promoters: the GAB promoter has an extremely high content while, in sharp contrast, no CpG islands are present in the LGA promoter (Fig. 6). Finally, the methylation-dependent immunoprecipitation of DNA and sequencing (MeDIP-seq) data for GAB and LGA regulatory regions in human brain also yielded a differential pattern: enhancer and alternate promoter regions for LGA do not show DNA methylation, while the proximal GAB promoter appears methylated (Fig. 6).

### Quantification of GAB and LGA Transcript Levels by Real-time PCR

**Figure 7:**
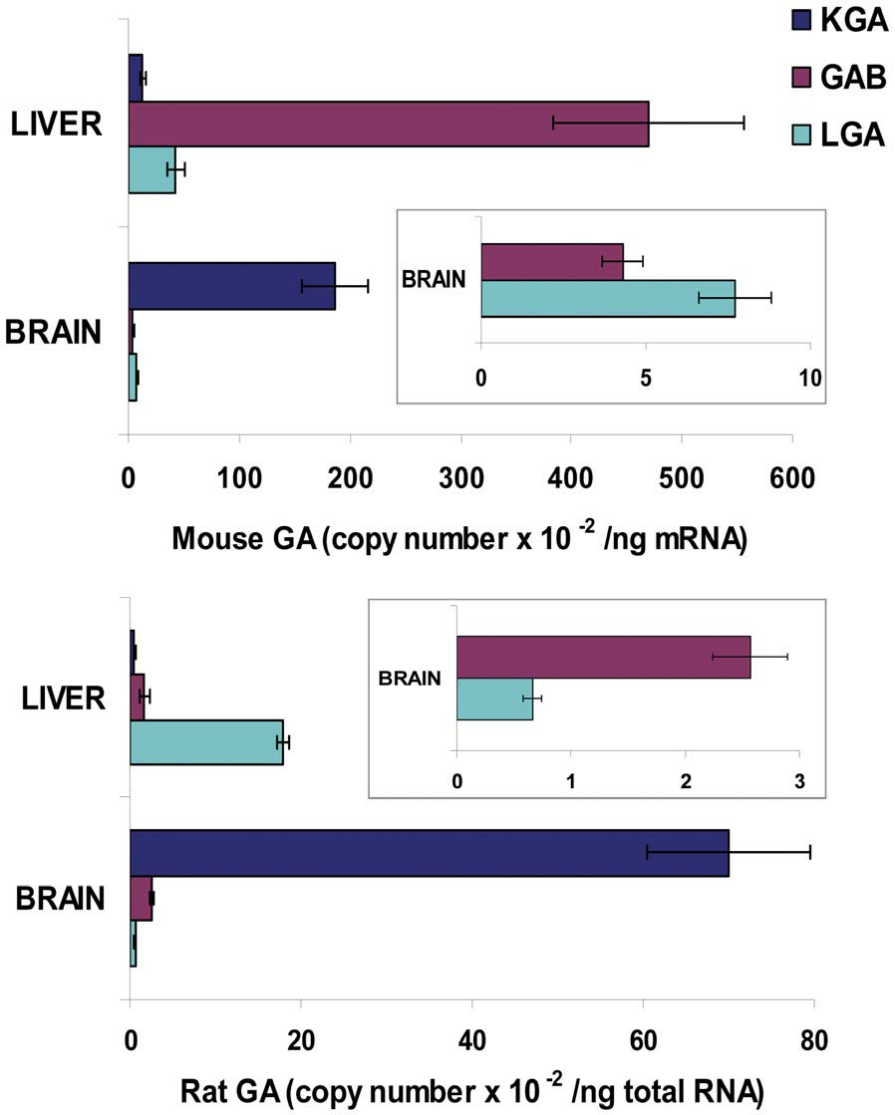
Quantification of mRNA levels of KGA, GAB and LGA transcripts in rat and mouse tissues by qPCR. The histogram shows the absolute copy number of mRNA transcripts for the KGA (dark blue), GAB (pink) and LGA (light blue) isoforms. Real time RT-PCR was performed as indicated in the Materials and Methods section. Results are mean ± S.E.M. of three independent experiments done in triplicate. Values were determined for liver and brain tissues from rat and mouse and are shown as copy number per ng of total RNA. Insets: GAB and LGA mRNA values in mouse and rat brains are depicted without KGA mRNA levels to appreciate differences among them.

We devised a real-time PCR method for quantification of the two L-type GA transcript variants in brain and liver tissues. The relative abundance of both L-type transcripts was tissue-specific and, more surprisingly, also species-specific. Thus, while LGA was slightly more abundant than GAB in mouse brain, the opposite was found in rat brain where GAB mRNA was fourfold the amount of LGA (Fig. 7). As a reference and useful control of the qPCR method developed, we also quantify the copy number of the KGA mRNA transcript. In mouse brain, KGA abundance was 15 times higher than the sum of L-type transcripts (GAB + LGA), reinforcing the role of KGA as the major isoform in this tissue (Fig. 7). The number of copies of KGA was 46.96×10^4^±9.48×10^4^, while that of L-type GA (GAB+LGA) was only 3.02×10^4^±0.33×10^4^. Similar results were found in rat brain, where KGA was again the most abundant isoform with a copy number 21-fold higher than that of LGA+GAB transcripts (Fig. 7).

In contrast, liver tissue lacks significant amount of KGA transcript and, therefore, it became a good control for our qPCR assay. The abundance of L-type transcripts in liver was absolutely dependent on the species being analyzed. Thus, while GAB was the predominant isoform in mouse liver, showing an 11-fold higher abundance than LGA, short transcript LGA was five-times more abundant than GAB in rat liver (Fig. 7). This strikingly different expression pattern observed for GLS2 transcript variants was unexpected and implies selective transcriptional regulatory mechanisms being operative in distinct tissues and mammalian species.

### Expression of *Gls2* Protein Isoforms: *in vitro* Transcription/translation and Immunoblot Analysis

Human LGA transcript possesses two in-frame ATGs in the first exon which are lacking in the orthologous transcripts from mouse and rat (Figs. 2 and 3). Both ATGs are surrounded by a sequence (CCCATGT and GGCATGC) at positions 83 and 218 of their respective mRNAs (Fig. 3), which match reasonably well with the Kozak consensus sequence for an optimal translation initiation in eukaryotes [35]. Interestingly, the Kozak sequence for the second ATG of human LGA is identical to that present in the long GAB transcript variant [15]. The open-reading frames (ORFs) beginning at the first two ATGs of human LGA encode putative proteins of 556 and 526 amino acids, respectively, with theoretical molecular masses of 56 and 51 kDa.

**Figure 8:**
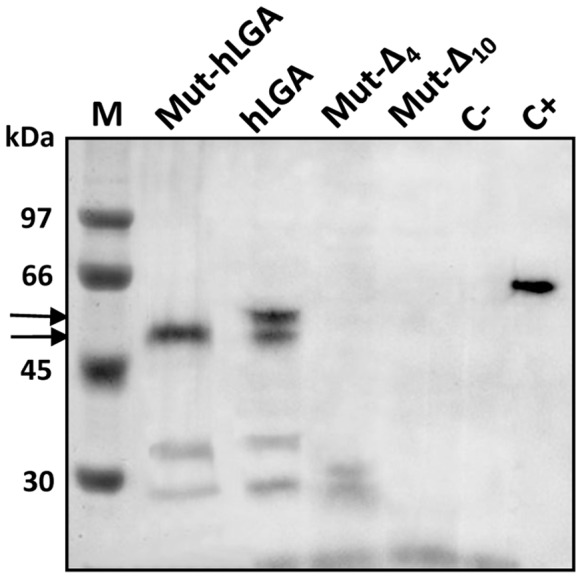
In vitro transcription and translation of human LGA cDNA. The ORF of human LGA (hLGA), deletion mutant hLGA (Mut-hLGA) starting at the second ATG, and Δ4- and Δ10-LGA deletion mutants were cloned into the pGEM-T vector and transcribed and translated *in vitro* in the presence of ^35^S-Met. The reaction mixtures were then analyzed by SDS-PAGE and autoradiography. Lane M, standard protein markers with the relative positions of prestained molecular mass markers indicated on the left; lane Mut-hLGA, aliquot of the translation mixture using the human LGA cDNA starting at the second in-frame ATG; lane hLGA, full-length human LGA; lane Mut-Δ4, aliquot of the translation mixture using the Δ4-LGA deletion mutant cDNA; lane Mut-Δ10, aliquot of the translation mixture using the Δ10-LGA deletion mutant cDNA; lane C-, negative control (pGEM-T with the hLGA cloned in the antisense orientation); lane C+, luciferase positive control. The radioactive LGA protein bands are indicated on the left using black arrows.

The proteins encoded by the cloned human LGA cDNA were demonstrated using the TnT® T7 *in vitro* transcription/translation system. We cloned the whole coding cDNA sequence of human brain LGA into the pGEM-T vector. Using a reticulocyte lysate system, the *in vitro* transcribed and translated [^35^S-Met]-LGA protein was analyzed by SDS-PAGE and autoradiography: two major bands were clearly seen at molecular masses compatible with proteins coded from alternative translation start ATGs (Fig. 8). Human LGA transcript variants lacking exon 4 and exon 10 were included as controls, as well as hLGA cloned in the antisense orientation: no relevant protein bands were detected (Fig. 8). In order to assure that both bands in the autoradiography correspond with proteins translated from the first two start codons, and not with shorter polypeptide products from downstream ATG codons, we prepared a deletion mutant construct of human LGA starting in the second ATG codon of the full transcript. Transcription and translation of this mutant cDNA yielded a clear protein band fully coincident with the lower band of the doublet seen for the LGA full transcript (Fig. 8). Thus, we confirmed that proteins translated from the two first start codons of the human LGA transcript can be obtained in roughly similar yields by using an *in vitro* reticulocyte system.

**Figure 9:**
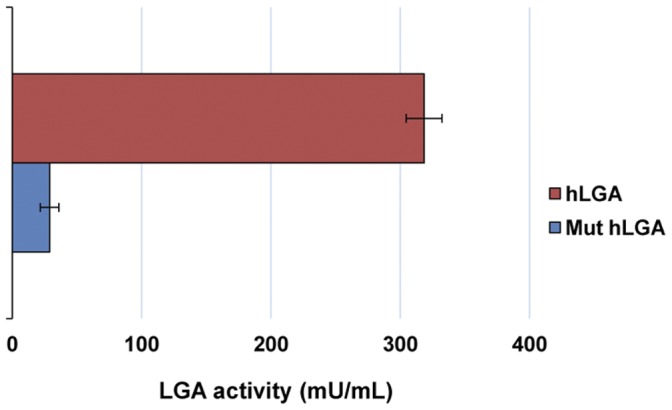
Glutaminase activity assay. The *in vitro* transcribed and translated human LGA (hLGA) protein and a deletion mutant (Mut hLGA) protein, starting at the second ATG initiation codon, were assayed for GA activity as described in the Materials and Methods section. Results are mean ± S.E.M. of three independent experiments: protein hLGA (pink bar); protein Mut hLGA (blue bar). Background activity was measured by using the pGEM-T[hLGA] construct cloned in the antisense orientation and was always subtracted from activity values obtained with the hLGA proteins. Values are expressed as milliunits of enzyme activity per ml.

Then, we tried to discriminate whether any of the LGA proteins produced *in vitro* was catalytically active. To answer this issue, cold aliquots of the *in vitro* translated proteins were evaluated for enzymatic activity. The reticulocyte lysate was clarified by ultrafiltration to remove potential contaminants that may interfere with the assay; furthermore, the buffer composition and pH of the translation mixture were also changed to reach optimum values for L-type GA [29]. GA activity was consistently detected well above control and background levels when assays were done with translated products from human LGA full transcript. The GA activity account for 318.50±13.84 mU/ml (nmoles of glutamate produced per min and ml of extract), the mean value for three different transcription/translation mixtures assayed (Fig. 9). However, the mutant LGA construct, starting at the second ATG codon of the ORF, only yields a residual near background GA activity (Fig. 9), even though it was satisfactorily translated (Fig. 8).

**Figure 10:**
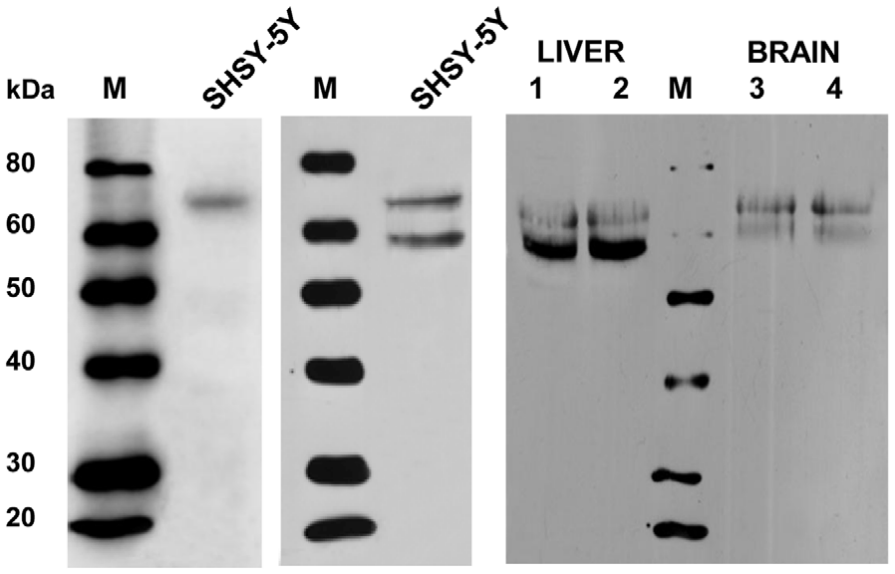
Immunoblot analysis of human SHSY-5Y neuroblastoma cells and rat liver and brain mitochondria. Cell extracts of human neuroblastoma SHSY-5Y cells (left and center panel) and mitochondria isolated from rat liver and brain (right panel) were analyzed by SDS-PAGE and Western blotting. Blots were revealed by chemiluminescence using polyclonal antibodies raised against the exclusive first exon of human GAB protein (left panel) or against the whole human GAB protein which recognize both GAB and LGA proteins (center and right panel). M, lanes containing the molecular mass markers indicated on the left margin; SHSY-5Y, lanes loaded with protein extracts isolated from human neuroblastoma cells; Liver 1 and 2: lanes containing total liver protein extracts and mitochondrial protein extracts, respectively; Brain 3 and 4: lanes containing total brain protein extracts and mitochondrial protein extracts, respectively.

Finally, the existence of LGA and GAB transcript variants in mammalian tissues was also assessed at the protein level by immunoblot analysis. The presence of protein products encoded by both alternative transcripts was revealed by using isozyme-specific antibodies against human L-type GA [27], which are able to recognize both GAB and LGA protein isoforms, and polyclonal antibodies monospecific for GAB protein raised against a synthetic peptide from exon 1, an amino acid sequence belonging exclusively to the GAB isoform. Human neuroblastoma SHSY-5Y cells express both GLS2 transcript variants (as checked by qPCR analysis, results not shown) and were used as a control for antibody validation in mammalian tissues. Two bands of the expected molecular masses for GAB and LGA peptides were revealed with the antibody recognizing common epitopes of both isoforms in neuroblastoma cell extracts (Fig. 10, central panel). Accordingly, the lower molecular mass band disappeared when a similar blot was revealed with GAB-specific antibodies (Fig. 10, left panel). Then, we assayed rat liver and brain mitochondria: a doublet of protein bands, with molecular masses consistent with GAB and LGA polypeptides, was seen in rat liver probed with antibodies recognizing both isoforms (Fig. 10, right panel). Interestingly, the lower LGA band displayed a stronger intensity than GAB, in agreement with the qPCR results of rat liver (Fig. 7). Finally, in rat brain mitochondria the lower LGA band was barely detected and only the GAB protein was revealed (Fig. 10, right panel), a result consistent with the relative transcript abundance determined by qPCR in rat brain tissue (Fig. 7).

## Discussion

Isoform-specific RT-PCR in brain and liver of three mammalian species –human, rat and mouse- demonstrates co-expression of two GLS2 transcript variants showing alternative first exons (Figs. 1 and 2): a long GAB transcript, previously cloned from ZR-75 breast cancer cells [15], and a short LGA transcript, lacking the first exon of GAB, and first characterized in rat liver [13], [33]. When we carried out these initial RT-PCR experiments, the *Gls2* gene in the rat and mouse genome databases had 17 exons and neither of them contained exon 1. The current version of genomic sequences for *Rattus norvegicus* strain BN/SsNHsdMCW (RGSC_v3.4) and *Mus musculus* (Ensembl mouse database, NCBIm37 sequence assembly) include transcript variant GAB based on similarity searches with human GAB, human *GLS2* gene and available EST. Experimental evidences obtained by RT-PCR in this study clearly support that GAB and LGA transcripts are co-expressed in liver and brain tissue from human, mouse and rat.

LGA protein isoforms display a very high degree of homology in human, mouse and rat, showing more than 91% of identity in their amino acid sequences. As paralogous genes, *Gls* and *Gls2* are presumed to have been derived by gene duplication followed by gradual changes in the sequences of both copies. Analysis of the coding sequences of human GA genes suggests that they have evolved from a common ancestral gene [34], as was previously noted for the rat genes [33]. Interestingly, paralogous proteins may acquire different functions during their evolution while maintaining a strong similarity in sequence and three-dimensional structure. In contrast with the GLS isoforms KGA and GAC, which mostly differ in their C-terminal regions, the only differences in GLS2 isoforms appear at the N-terminal region. Interestingly, the human LGA isoform is longer than its mouse and rat counterparts, showing an extension of 30 amino acids at the N-terminal end: 24 novel amino acids followed by the first 6 amino acids of exon 2 of human GAB (Fig. S1). The N-terminal region of GA is related with subcellular location and posttranslational processing [28]. Bioinformatics analysis of human LGA protein does not find a consensus mitochondrial targeting sequence at its N-terminus; however, a secretory signal peptide was predicted for the first 16 amino acids of the LGA protein translated from the second ATG [36].

Cloning of the human LGA transcript (Fig. 3) allowed the discovery of the transcriptional mechanism regulating GLS2 transcript products. Primer extension analysis revealed the LGA transcription start site, mapping to the 3′ end of intron 1 of the *GLS2* gene (Fig. 4). This site showed great sequence identity with the TSS of rat liver LGA. In addition, TATA-like and CAAT boxes were present at consensus distances from this transcription initiation site. The cloning and primer extension data for human LGA unambiguously demonstrate that this isoform arises by alternative TSS of the *GLS2* gene.

The location of the LGA alternative transcription start point in intron 1 immediately suggests the presence of an alternative promoter for this isoform, because the GAB canonical promoter is more than 7 kb apart from the TSS of human LGA. As expected, the last 1,5 kb of intron 1 has many transcription factor recognitions boxes. Of note, three DNA binding consensus sequence for p53 in the human *GLS2* gene were recently found: one in the canonical GAB promoter and two others in intron 1, reinforcing the view that this is a true promoter for the LGA transcript [24]. Moreover, bioinformatics analysis of intron 1 yields potential recognition binding sites for AP-1, GATA-1, C/EBP and SP-1 in the near promoter region and also detects a distal regulatory enhancer region almost 7-kb away from the TSS (Fig. 5). Sequence analysis of alternative and canonical human *GLS2* promoters did not show significant homology between them. The promoter of the orthologous rat LGA transcript was previously cloned and sequenced (GenBank# L76175) [33]. Nowadays, it can be mapped at the end of intron 1 of the rat *Gls2* gene, approximately 1.1 kb upstream of exon 2 in the current version of rat chromosome 7 (Rat Genome Database, v3.4, http://rgd.mcw.edu/). Taken together, experimental and bioinformatics data point toward the same conclusion: LGA arises by direct transcription of the *Gls2* gene from an alternate promoter located at the end of intron 1.

Canonical and alternate *GLS2* promoters differ in basic elements like CpG islands (Fig. 6). This difference has been noted previously as a kind of hallmark for alternative promoters, whose frequency of CpG islands was less than half of that seen in normal canonical promoters [7]. In addition, it has been shown that CpG-containing promoters have a ubiquitous expression [37], whereas almost a triple number of alternative promoters, on average, show tissue-specific or signal-dependent expression instead of being ubiquitously expressed [7]. Therefore, the distinct CpG content paves the way for a differential regulation of both transcripts by epigenetic mechanisms. Furthermore, the pattern of methylation of both promoters is quite different (Fig. 6). The existence of separate methylated and unmethylated promoters for the same human gene has been recently highlighted as a mechanism for regulation of alternative transcription in normal differentiation and cancer, allowing expression of a gene even though its canonical 5′-promoter remains methylated [38], [39]. Thus, DNA methylation may control alternative promoter usage.

Although alternative promoter use was first described in 1981 [40], its relevance for generation of protein and regulatory diversity has not been fully appreciated until quite recently. The use of alternative promoters does not always result in the production of different proteins; in fact, it appears that 60–80% of genes with alternative promoters produce transcripts with identical ORFs [41]. A recent study for alternative promoters of human genes using exon-microarray data identified 7,708 genes with alternative promoters, of which 57% transcribed a common first exon and only 42% transcribed a distinct alternative first exon; in all cases, the alternative promoter was fully embedded in an intron [42]. In the case of *GLS2*, the alternative promoter is fully embedded in intron 1, yields a transcript with a distinct first exon and translates a protein isoform with different N-termini (LGA). Thus, *GLS2* has a novel intronic CpG-less promoter and, therefore, must be added to the ongoing annotation and expanding list of alternative promoters of human genes. It is noteworthy that human genes with distinct alternative first exons possess a 2-fold higher occurrence of promoters lacking CpG-island in one of their TSSs [42], exactly as happens with *GLS2*.

Interestingly, previous work has found that brain is one of the tissues where the presence of alternative promoters of human genes was most abundant [7]. The same authors reported that about 52% of the total human genes were subject to regulation by putative alternative promoters, and genes encoding signal-transduction related proteins were more likely to display alternative promoters. It is tempting to speculate that GLS2 transcripts may have this kind of regulatory mechanism in brain because the function of GAB and LGA transcripts might be associated to signaling or regulatory functions, besides their role as glutamate-producer enzymes [28], [43].

Another posttranslational mechanism recently discovered is nonsense- mediated mRNA decay (NMD). Large-scale analysis of alternative isoforms of known human genes found that one-third of the alternative transcripts examined contained premature termination codons [44]. The authors concluded that coupling of alternative splicing and NMD is an important mechanism of regulating protein expression. Interestingly, both GA human genes were found to be targeted for NMD in this study: two NMD-candidates were reported for *GLS2* and one for *GLS* [44]. Here, we have annotated two more non-coding RNAs for human *GLS2* (lacking exons fourth and tenth, respectively) and one for mouse *Gls2* (partially retained intron), supporting the view that NMD may play a functional role in regulating GA protein expression levels.

The final aim of our study on transcriptional regulation of the *Gls2* gene will be a full description of the *in vivo* function of both alternative products. Actually, alternative promoter may play a role in generating species-specific diversity, as can be inferred from our results on relative abundance of GAB and LGA in rat and mouse tissues. An unexpected result was the dramatic change in transcript levels observed in liver: GAB was the most abundant isoform in mouse (11-fold *vs* LGA), but not in rat where LGA predominates over GAB almost in the same proportion (10.5-fold). In brain, the pattern of mRNA expression was the opposite of that previously found in liver: LGA was the main isoform in mouse while GAB showed the greatest abundance in rat. In rat and mouse brain, KGA was clearly the predominant isoenzyme accounting for more than 90% of total GA transcripts. As far as we know, this is the first report quantifying the mRNA levels of main GA isoforms (KGA, GAB and LGA) in whole brain of mammals.

Alternative mRNA isoforms not always translate into different protein isoforms and can be involved in regulation of translation initiation, RNA editing and other processes. Therefore, we searched for additional experimental evidences at the protein level to assess the functionality of GLS2 mRNA variants. When the cloned human LGA cDNA was *in vitro* transcribed and translated two protein bands labeled with [^35^S]-Met were clearly seen. By running a parallel deletion mutant construct, with its ORF starting at the second in-frame ATG of the LGA transcript, we were able to conclude that both bands correspond to products arising from the first two in-frame ATG of its sequence. When activity assays were set using the *in vitro* translated products, GA activity was consistently detected for the whole LGA cDNA construct but not for the deletion mutant. This result agrees with the fact that full-length LGA protein, starting at the first ATG, was the only product displaying catalytic activity, even though protein translation may start from any of the two initial ATGs with roughly equal efficiency, at least in the rabbit reticulocyte lysate system. Taken together, the results point toward the existence of only one functional human LGA: the protein translated from the first start codon of its mRNA sequence.

On the basis of differences in molecular structure, kinetic behavior and subcellular location we first concluded that human GAB should be a new GA isozyme and not the human orthologous of the rat liver enzyme [16], [29]. Now, we have found conclusive experimental evidences supporting that hypothesis. Western blot results in rat liver tissue were consistent with co-expression of GAB and LGA proteins. Two main bands with apparent molecular masses compatible with GAB and LGA isoforms were detected with polyclonal antibodies against the whole GAB protein (Fig. 10). A similar result was previously obtained in mouse liver mitochondria [30]. To obtain further insights into their identity, we probed the blots with an isoform-specific polyclonal antibody that only recognizes the long GAB transcript: in this case, the lower molecular mass band disappears. This result is in accordance with GAB being the protein with higher molecular mass and LGA the lower band in the doublet detected in rat liver. The two GLS2 peptides from the brain appear to differ in size somewhat from the peptides in the liver, probably reflecting a different pattern of posttranslational modifications. Interestingly, a similar behavior was already detected in mouse liver and brain mitochondria [30]. In summary, the protein expression results support those obtained at the mRNA level and suggest a parallel behavior between transcript abundance and protein concentration for each GLS2 isoform.

Our finding of alternative L-type transcripts by use of alternate promoters and consequent alternative use of first exons for the mammalian *Gls2* gene may explain most of the previous experimental data suggesting the existence of two L-type isoforms. Further studies will be needed to detail the *in vivo* function of the alternative products of the mammalian *Gls2* gene. Work is in progress to fully characterize the transcriptional and posttranscriptional regulation of GAB and LGA transcripts and their relevance in cancer and other diseases.

## Supporting Information

Figure S1 **Comparison of N-terminal amino acid sequences of LGA and GAB proteins from mouse, rat and human tissues.** Sequence alignment of the N-termini of the indicated proteins were done using Multalin program (http://multalin.toulouse.inra.fr/multalin/). For the sake of clarity, the same order as in the nucleotide sequence comparison shown in Fig. 2 was maintained. Top panel: amino acid sequences of mouse brain and human liver LGA were aligned with the sequence of rat liver LGA; bottom panel: amino acids of rat liver and mouse brain GAB proteins were aligned with the N-terminal sequence of human GAB from ZR-75 breast cancer cells. Identical amino acids are indicated in red, different amino acids are labeled in blue.
(TIF)

Table S1 **Oligonucleotide sequences used for real-time RT-PCR of GAB- and LGA-specific amplicons in brain and liver tissues from rat and mouse.** The nucleotide sequences for sense (forward) and antisense (reverse) primers used in the real-time RT-PCR experiments are shown. Mouse primers are indicated with the abbreviation “m” and rat primers with “r”. Primers employed for the house keeping genes β-actin and Rpl19 are also shown. The size of the expected amplified products (in base pairs) and their molar masses (g/mol) are displayed in the last column.
(TIF)
