# Supplementary figures and images for: Mammalian Glutaminase Gls2 Gene Encodes Two Functional Alternative Transcripts by a Surrogate Promoter Usage Mechanism
Source: PLoS One. 2012 Jun 5;7(6):e38380. doi: 10.1371/journal.pone.0038380 (PMC3367983; doi:10.1371/journal.pone.0038380)

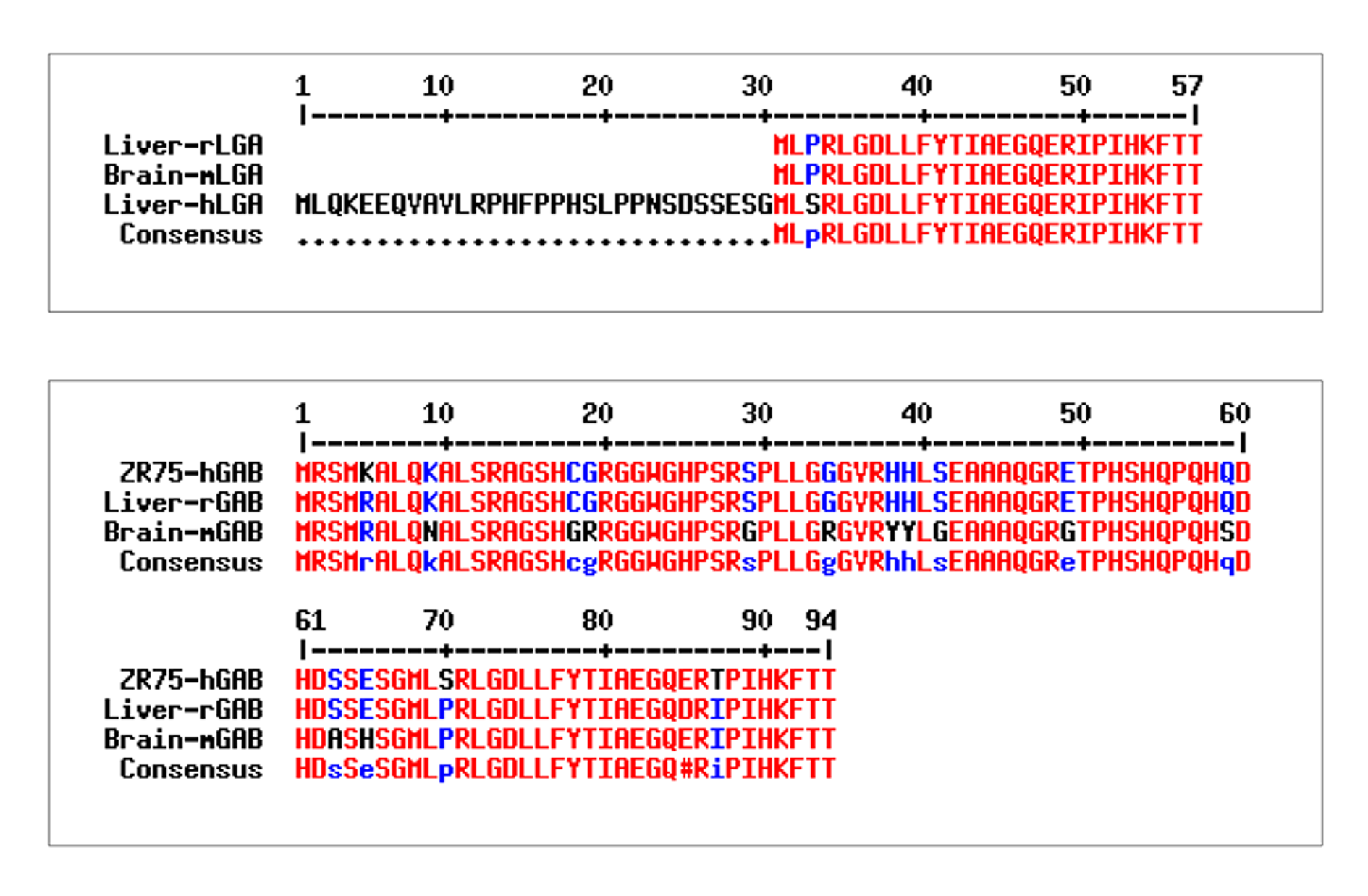

Supplement: Figure S1 — Comparison of N-terminal amino acid sequences of LGA and GAB proteins from mouse, rat and human tissues. Sequence alignment of the N-termini of the indicated proteins were done using Multalin program (http://multalin.toulouse.inra.fr/multalin/). For the sake of clarity, the same order as in the nucleotide sequence comparison shown in Fig. 2 was maintained. Top panel: amino acid sequences of mouse brain and human liver LGA were aligned with the sequence of rat liver LGA; bottom panel: amino acids of rat liver and mouse brain GAB proteins were aligned with the N-terminal sequence of human GAB from ZR-75 breast cancer cells. Identical amino acids are indicated in red, different amino acids are labeled in blue. (TIF) [file pone.0038380.s001.tif]

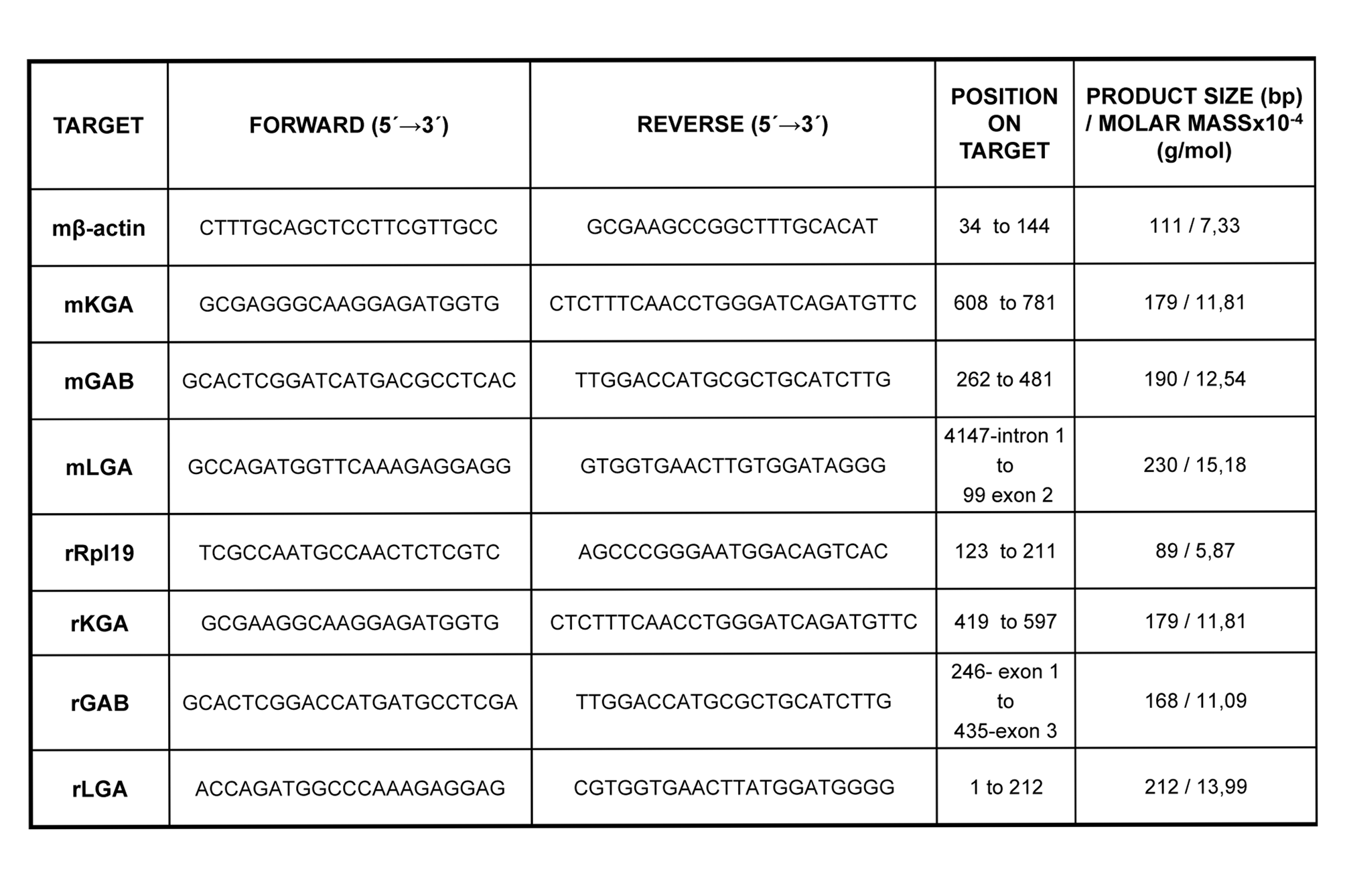

Supplement: Table S1 — Oligonucleotide sequences used for real-time RT-PCR of GAB- and LGA-specific amplicons in brain and liver tissues from rat and mouse. The nucleotide sequences for sense (forward) and antisense (reverse) primers used in the real-time RT-PCR experiments are shown. Mouse primers are indicated with the abbreviation “m” and rat primers with “r”. Primers employed for the house keeping genes β-actin and Rpl19 are also shown. The size of the expected amplified products (in base pairs) and their molar masses (g/mol) are displayed in the last column. (TIF) [file pone.0038380.s002.tif]
